# Increasing participation of underrepresented groups in cancer early detection research: a scoping review

**DOI:** 10.21203/rs.3.rs-5005757/v1

**Published:** 2025-10-02

**Authors:** Frederike Brockhoven, Maya Raphael, Nora Pashayan, Ignacia Arteaga

**Affiliations:** University of Oxford; NIHR Bioresource, University of Cambridge; University of Cambridge; University of California, San Francisco

**Keywords:** minoritised groups, diversity, early detection, cancer research, research culture, research participation

## Abstract

**Background:**

Improvements in access to early-detection research on cancer are still urgently needed to ensure that new research on early-stage cancer detection benefits all groups in society. To achieve this, cancer early detection (ED) studies must include participants from all walks of life. There are unique aspects to cancer early detection research that may deter potential research participants and complicate efforts to involve people from underrepresented backgrounds that require a review on its own merit. For instance, a unique risk for cancer ED research is overdiagnosis and overtreatment, in which a tumour is uncovered and treated that would not have led to the patient’s death if left undiscovered and untreated. This potential ‘side effect’ of cancer ED research participation is particularly problematic for those without adequate access to healthcare and insurance.

**Methods:**

Contributing to a growing literature on approaches to increase diversity in cancer detection research, we conducted a targeted scoping review to identify published approaches that have been implemented and tested, either quantitatively or qualitatively, to improve participation of minoritised groups in cancer ED research.

**Results:**

This review identifies themes in the 38 included studies that aimed to recruit and involve participants from underserved groups in cancer ED research so that future studies may learn from or further test these varied strategies. We narratively group the review in terms of the barriers identified, and the approaches that have been designed to improve participation. These include rethinking recruitment locations and partnerships with local communities, designing educational interventions, combining research with community needs, increasing cultural competence of research teams, and overcoming practical barriers in study design.

**Conclusions:**

This scoping literature review highlights various tools, empirically tested, that research teams can employ to improve participation rates of groups underrepresented in cancer ED research. Combinations of these methods can help overcome the perceived barriers to participation in cancer research that mainly affect people without a cancer diagnosis from these minoritised groups. Not only will these methods increase the generalizability and representativeness of studies; the highlighted approaches also contribute to a more significant shift in research culture towards less extractive and more trusting relationships between researchers and the public.

## 1 INTRODUCTION

Early detection (ED) and cancer diagnosis are vital for improving the prospects of cancer patients. When cancers or pre-cancerous lesions are detected earlier, patients’ chances of surviving their disease are dramatically higher(2). Hence, cancer ED research has become a priority of cancer research in the United Kingdom(1), the United States(3), and other countries(4). Yet, improvements in access to early-detection research on cancer are still urgently needed to ensure that new research on early-stage cancer detection benefits all groups in society(1). To achieve this, cancer ED studies must include participants from all walks of life. Still, similarly to many other health research fields, people from ethnic minorities and other underserved and minoritised groups are usually underrepresented in cancer ED studies(5–7).

There are unique aspects to cancer early detection research that may deter potential research participants and complicate efforts to involve people from underrepresented backgrounds (8, 9). Firstly, cancer ED research usually requires the participation of participants who are cancer free or asymptomatic, which thus requires ‘healthy’ people to consider the possibility of developing cancer. Having to think about a potentially lethal disease can put people off participation, especially those who already struggle to access adequate healthcare. This makes participation in cancer ED research a different recruitment process from other cancer clinical trials, which can recruit cancer patients already being treated in hospitals who are aware of their condition. Secondly, cancer-free and symptomatic people may be deterred by the invasive participation procedures that cancer ED research might require (10). Lastly, participating in cancer ED research can sometimes lead to the discovery of health problems, such as cancer or a higher risk of developing one. Overdiagnosis and overtreatment of slow-growing or indolent cancers; and detection of early cancers for which a treatment has not yet proven effective are known risks (11–14). Again, this potential ‘side effect’ of cancer ED research participation is particularly problematic for those without adequate access to healthcare and insurance (9) .

These aspects of cancer ED research demonstrate the need for a scoping literature review about strategies to involve underrepresented groups in research in this particular field of cancer research. Although insights from systematic literature reviews and scoping reviews focusing on increasing participation of minoritised groups in cancer clinical trials (15–21) or clinical trials for other conditions(22–24) are beneficial, this scoping review seeks to map out only those tested interventions suitable for the involvement of underrepresented groups in the unique processes of cancer ED research.

This review is part of a wider effort to develop recommendations for improving diversity and trust-building in cancer ED research. As part of ‘REPRESENT: A Community Engagement Roadmap to Improve Participant Representation in Cancer Research Early Detection’(8, 9), we conducted a targeted scoping review to identify the approaches that have been implemented to improve participation of minoritised groups in cancer ED research. The method of a targeted scoping review was chosen to map and analyse the wide range of interventions that have been tested, without excluding studies based on small sample sizes or heterogeneous evaluation methods(25). This review identifies themes in the 38 included studies that aimed to recruit and involve participants from underserved groups in cancer ED research so that future studies may learn from or further test these varied strategies.

## 2 METHODS

### 2.1 Search strategy

The databases used for this systematic literature review, conducted in April 2022, were PubMed and PsycINFO. The question we sought to answer is: ‘What approaches have been implemented to increase participation of people without a cancer diagnosis from underrepresented groups in cancer early detection research?’. The search strategy consisted of four themes, each forming a set of related (MeSH) terms: research (e.g., clinical trial), underrepresented communities (e.g., underserved, minorit*, African American, Black, Latin*), participation (e.g., engagement, involvement, recruit*), and cancer (e.g., oncol*, lymphoma). The category of ‘underrepresented communities’ included the highest number of terms to ensure a wide range of underserved groups were included in the search. These groups included various ethnic, indigenous, and linguistic minorities, gender and sexual minorities, people with physical disabilities and neurodiverse people, groups marginalised based on their geographic location or socio-economic status, and those often excluded based on their immigration status. Since there are also various terms to denote participation, each with a nuance that can differ between countries and research contexts, it was decided to include all terms that indicate some level of involvement in research. Search strategies and filters were modified for the corresponding search database.

Inclusion criteria for the review were the following: 1) only published empirical and peer-reviewed studies; 2) studies published between 2002 and April 2022; 3) research undertaken in a High-Income Country; 4) studies involving adult research participants (18 years or above); 5) studies directly related to cancer early detection research; 6) studies involving participants who have *not* yet been diagnosed with cancer; 7) research that is not primarily therapy-focused; 8) articles aiming to enhance the recruitment and participation of underserved groups; 9) studies that implement and intervention to improve recruitment or participation in cancer studies *and* quantitatively/qualitatively evaluate its effectiveness; and 10) articles published in English.

**Figure 1:**
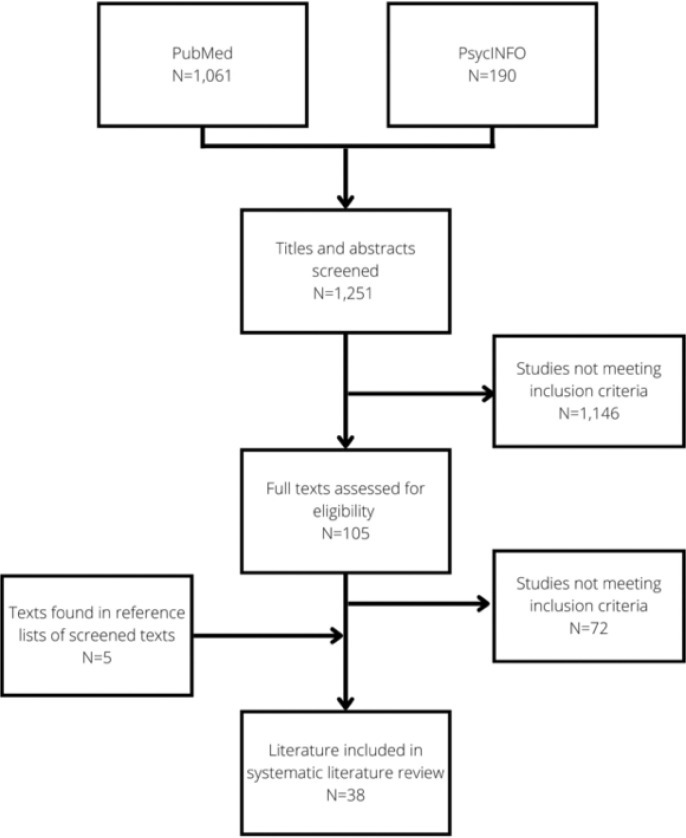
Flow chart of the review inclusion/exclusion process.

The search yielded 1,061 results on PubMed and 190 results on PsycINFO, whose titles were all scrutinised to filter out irrelevant papers. The abstracts of all remaining studies were read to select those that may fit the inclusion criteria. Figure 1 shows a flow chart detailing manuscript inclusion. Two investigators determined manuscripts’ eligibility independently: one author (FB) screened and read all identified papers, and another (MR) independently screened a 25% sample of the 1,251 identified studies. Given that the agreement on inclusion and exclusion was satisfactory, FB screened the rest of the papers alone. This process generated a sample of 33 articles, to which five more articles found in references of screened documents were added. This led to a final sample of 38 papers. Figure 1 presents the flow chart describing the selection process.

### 2.2 Analysis

An iterative process created a standardised method for extracting information from the eligible papers. We identified frequencies and means, where possible, and coded essential and detailed study characteristics in Microsoft Excel. In addition to the basic information of the article (title, authors, date), data was retrieved on the targeted population, geographic location of participants, barriers to research participation identified, cancer type focused on, sample size, and intervention(s) implemented, among others. Retrieved manuscripts were then analysed to identify themes and to synthesise tested interventions to improve the participation of groups often underrepresented in cancer early detection research. The quality of the included studies was also assessed using CASP guidelines(26), which included attention to potential risk of bias.

## 3 RESULTS

### 3.1 Characteristics of included articles

Table 1 presents the thirty-eight included manuscripts published between 2004 and 2022, including their approaches, identified barriers to participation, social group they focused on, type of cancer (if any), evaluation methods and risk of bias.

Due to the inclusion criteria of the study, the final selection of studies was overwhelmingly carried out in the United States, except for one study in the UK (27). In addition, studies often focused on similar minoritised groups: African American(10, 27–51) and Latinx or Hispanics (30, 35, 36, 43, 48, 49, 52–56). However, category assignment was not exclusive, as some studies usually included multiple underrepresented groups simultaneously in their intervention.

Those studies that sought participation in research on specific cancer types predominantly focused on breast (10, 28, 32, 45–47, 50, 52–54, 57–59), colorectal (31, 33, 35, 41, 44, 48, 57, 59), prostate (27, 33, 34, 36, 38, 39, 41, 46) and lung (33, 36, 41) cancers, sometimes in combination. However, no cancer type was specified in many cases, or the study could encompass multiple cancers. This was the case of studies focusing on enhancing diversity in biospecimen collection (40, 43, 49, 60).

The number of participants in the selected manuscripts that reported this number ranged from 17 to 154,934 per study. Yet, not all articles reflect the total number of enrolled participants: in some reports, these participants were divided over different phases of the project, whereas in other manuscripts that analyse several distinct studies, the participants are divided between those studies.

Of note, there were two cases of overlap between the included manuscripts. Greiner and colleagues (35) analysed multiple research projects, including a study described in a dedicated separate manuscript first published by Friedman and colleagues (34). To avoid duplication in our analysis, we include Greiner’s study only when it focuses on the remaining four studies not accounted for in (14) and refer to Friedman and colleagues’ study otherwise. Moreover, Sheppard and colleagues’ report (59) describes complementary details about recruitment strategies and results to those included in Kreling and colleagues’ (53) manuscript. We include both manuscripts in this case: Kreling and colleagues (16) account for and measure the effectiveness of the broader research infrastructure they have put in place to serve the Latinx population in their catchment area. At the same time, Friedman’s work provides a narrower descriptive report of recruitment methods utilised in the research coalition.

In the following pages, we narratively describe the barriers to participation identified in retrieved articles, to then unpack the interventions that were tested as part of research teams’ efforts to improve participation of underserved groups. We divide those approaches in: rethinking recruitment locations and partnerships with local communities; designing educational interventions and including peer-educators; combining research with community needs; increasing cultural competence of research teams; and overcoming practical barriers in study design.

### 3.2 Barriers to cancer ED research participation

Each included study identified barriers to participation for underrepresented groups based on original research or existing literature. These identified barriers informed the rationale behind the intervention to increase research participation. Table 2 shows the frequency of the various obstacles identified in the sampled studies, with some studies identifying multiple barriers for the selected population.

Mistrust, the most cited barrier to cancer ED research participation among underrepresented groups, could refer to distrust of both healthcare and biomedical research (27, 29–31, 33, 34, 39, 46, 47, 51, 58, 61). Mistrust in healthcare and biomedical research can stem from historical wrongdoings, such as the infamous and widely cited Tuskegee Syphilis Study (62), as well as ongoing experiences of exclusion and discrimination in healthcare settings (51). Mistrust was coded separately from concerns about confidentiality, such as data breaches or information being passed on (29, 43, 60, 61), or privacy issues (29, 60), and fear of the clinical procedures one might undergo as part of cancer ED research, such as venipuncture or x-rays (28, 29, 40, 49, 60).

Perceived lack of knowledge of cancer, cancer early detection, and cancer ED research was also a commonly cited reason for nonparticipation (28, 30, 34, 35, 38, 41, 46, 49, 53, 55, 56, 58, 63, 64), as well as a lack of awareness that there are cancer ED trials that healthy people can sign up for (28, 30, 31, 34, 38, 39, 41, 43, 48, 52, 55, 64). While these barriers were often framed as a problem within the underrepresented community, we know that they can also be interpreted as a failure by research institutions to reach these groups, if not a failure of wider society to teach everyone basic knowledge about cancer and cancer research (55, 65)—a much smaller number of studies (32, 35, 43, 47, 54), placing the problem on the institutional side of the equation.

### 3.3 Approaches to improve participation of underserved groups in cancer ED research

The included papers displayed a high level of heterogeneity and often combined different recruitment and participatory strategies. Therefore, the following highlighted strategies should not be interpreted as distinct, singular solutions but rather as tools that can be employed in various combinations to improve the participation of underserved groups in cancer ED research.

#### 3.3.1 Recruitment locations and partnerships

With mistrust in clinical research and healthcare being one of the critical barriers to research participation, solutions for recruiting and involving people from minoritised groups in cancer ED research often focused on engaging potential participants in settings that inspire trust. This includes spaces with trusted ‘gatekeepers’: places of worship, healthcare centres, and barbershops. To approach people there for research participation, it thus uses ‘trust by proxy’, where the environment and people associated with it facilitate trust-building between research teams and potential participants.

##### Church

A remarkably high volume of studies predominantly chosen to reach Black participants focused on establishing partnerships with places of worship: churches (28, 29, 31, 33, 34, 36, 42, 44, 46, 53, 60) or, in one case, temples (57). Churches frequented by a particular minoritised group were approached by researchers and asked to either allow recruitment via the church’s communication channels or to set up a more comprehensive (community-based) programme combining long-term research with other activities (see section 3.3.3). A successful example is the study by McNeill and colleagues (42), who developed a partnership with three African American churches in Houston, one of them with as many as 15,000 members, for a large cohort study. The research team spent one year building a trusting relationship with the churches. In addition to these dialogues, research teams attended worship programmes, responded to requests for talks on cancer control, and guided church members to local screening services. The trusting, reciprocal relationship between the research team and the churches led to an overwhelmingly positive response to the recruitment efforts. Similarly, Slade and colleagues (46) developed a trusting relationship with the leadership of participating churches, who then helped researchers plan research that limited barriers to participation, for example, by scheduling events at the church around activities that would already draw people to church.

##### Barbershops and salons

Five studies used barbershops and beauty salons to reach potential participants from underserved groups for cancer ED research participation (31, 35, 39, 44, 45). Since barbershops and salons are often gender-segregated, they facilitate reaching people from a particular gender and provide an environment where a (sex-specific) cancer can be discussed without the presence of the other sex. In addition, Jones, Steeves, and Williams (39) note that barbershops have historically been one of the few places where ‘African American men, regardless of their education, socioeconomic status, or occupation, could congregate and voice their opinions about various topics’. Therefore, the barbershop is a location where conversations about cancer, which will affect many men in their lifetime, and research participation can be introduced.

##### Healthcare centres

Ten studies chose clinical settings as one of the recruitment sites (32, 35, 37, 40, 41, 48, 51–53, 57). This strategy could be a letter or message from a physician (48, 57), multiple callbacks from the clinic (32), or face-to-face recruitment at either a community-based or specialist clinic (12, 14, 17, 32, 34–35, 37). Tabriz and colleagues (48) attempted to recruit participants through an online patient portal, which would mitigate human bias in trial invitations. Still, they found that this recruitment method led to less ethnically diverse study samples as Black and Asian primary care patients were less likely than White patients to read the portal message, opening the attached link in the message, and consenting to participate in the trial. Similarly, in a study by Wang and colleagues (29), a bilingual, signed physician’s letter sent to Chinese Americans saw an enrolment rate of 32% on average, but this enrolment strategy enrolled comparatively fewer recent immigrants and uninsured Chinese Americans than community-based approaches. These findings illustrate that this recruitment approach risks excluding groups with less trust in healthcare institutions.

#### 3.3.2 Designing Educational Interventions and Including Peer-Educators

Since lack of knowledge about cancer ED and cancer ED research was identified as a significant barrier to research participation, interventions promoting education and awareness were often included as part of recruitment strategies. Fourteen studies organised educational sessions to counter stigma, stereotypes and false information about cancer research and associated data-collection procedures (10, 28, 29, 34, 38, 40, 41, 43, 47, 49, 53, 54, 56, 64), hypothesising that increasing the knowledge about cancer, early detection, and cancer ED research in communities would lead to higher participation rates. Smith and colleagues (47) found that educational workshops followed by question-and-answer sessions were the most successful recruitment strategy, the latter being essential to give participants space to get their questions answered instead of being passive recipients of information. Another practical approach among these studies was to ask those present at the educational session to participate in the data collection immediately afterwards. However, this type of intervention was only feasible when participants were asked to either sign up to study, donate a biospecimen sample, or complete another brief test or task (10, 28, 40, 43, 56). Four studies designed interventions through culturally tailored videos to raise awareness of biobanking and cancer screening research projects (27, 30, 56, 60).

##### Peer Educators

Seven papers from the selected studies recruited and trained peer educators from the target community to disseminate information about cancer (ED) and research and recruit potential study participants. Peer educators, described by various terms such as ‘*Promotores de Salud*’ (54, 55), ‘community health educators’ (30, 64) or ‘local champions’ (61), were chosen to disseminate information in a particular community because they are more likely to be considered a trusted source than a research team of ‘outsiders’, and because they are more likely to understand cultural sensitivities and appropriate manners of communication. Therefore, recruiting peer educators not only addresses lacunae in terms of knowledge of cancer, early detection, and research but also considers the lack of trust in biomedical research and healthcare professionals, as well as the importance of cultural appropriateness. In addition, peer educators can reach community members through their existing networks, which means they can get more isolated individuals (54).

Involving peer educators in study design and implementation also often seemed coupled with a desire to alter power dynamics between research teams and communities involved in cancer ED research. In contrast with one-off recruitment strategies, peer educators can be part of a long-term relationship between research teams and their communities and become essential actors in the bidirectional communication between the two sides.

Moreover, training peer educators (e.g., in presentation skills, leadership skills, health promotion and recruitment strategies) and investing in their development can be part of an effort to ‘give back’ to the researched community and to make the relationship between researchers and communities mutually beneficial. The *promotoras* participating in the study by Larkey and colleagues (54) developed skills and obtained experiences that led to further opportunities for career advancement.

#### 3.3.3 Combining research with community needs

People approached for cancer ED research may not see cancer ED research as a priority among all other causes and activities that require their time and attention. Moreover, participation in research activities can be an unappealing prospect. Therefore, seven studies in the sample combined research activities with community needs to make participation more appealing and to provide essential information to communities that could benefit from this. As a result, incorporating services that address community needs can render the researcher-participant relationship less extractive and contribute to a trusting relationship between the research team and research participants from marginalised communities.

##### Screening service and research participation

Three studies invited research participants by bringing screening services into the communities researchers hoped to recruit from (10, 28, 52). For example, McElfish and colleagues (52) recruited rural, medically underserved women in Arkansas (USA) for their breast cancer study using a mobile mammography unit (MammoVan). The unit provided breast cancer screening to women without a history of breast cancer free of charge, after which the women were asked to participate in the study, which involved an additional survey and saliva sample. The MammoVan recruitment strategy led to more rural and lower-educated participants (52). In the other two studies, potential research participants were offered several tests to collect health data, after which they were asked to donate these samples for research purposes. (10, 28)

This approach successfully reached underrepresented groups in cancer ED research in all three studies, but two things must be noted. Firstly, this method only has added value in areas where access to screening and other health services is insufficient: in places where potential research participants have easy access to cancer screening services and other health tests, this approach would have little added value for communities that researchers hope to recruit from. Secondly, the types of procedures offered to and asked from potential participants influence the likelihood of participation: Yeary and colleagues found that research activities with a higher participant burden, such as mammography and blood tests, saw much lower participation rates than simple procedures, such as surveys and anthropometric measurements, even when the indicated intent to participate was equally high or higher (10).

##### Educational needs and research participation

Rather than offering insight into personal health, four studies (41, 42, 46, 50) combined research recruitment with meeting the educational needs of a particular group or community. Educating about health topics can increase intent to participate in research (60), but offering educational sessions about various issues can also be part of building trust between researchers and participants and an act of service to a participating community. This is illustrated by a study conducted by Langford and colleagues (41), for which the research team organised breakfasts for African American men. In addition to talks about prostate cancer and cancer research, the team solicited ideas from men to address topics they wanted to learn about. As a result, some breakfast talks covered seemingly unrelated themes, such as self-motivation and estate planning, demonstrating the researchers’ recognition of the broad way in which men define health. By inviting various speakers, from NFL players to urologists, to the breakfast programme, the research team offered their resources to benefit the community.

#### 3.3.4 Increasing cultural competence of research team

The cultural competence of research teams and outreach materials was an overarching and often-cited theme in the selected studies. However, in six studies (29, 42, 50, 53, 57, 59), increasing the cultural competence, or cultural humility (66) of the research and recruitment staff, was a key strategy. In all cases, manuscripts mentioned having a diverse team that included members of the community the study sought to engage. In addition, some research teams were explicitly trained in cultural competence. For example, Wallington and colleagues (50) employed a highly diverse research and recruitment team, who were all trained in core practices of cultural competence, “acquiring and institutionalising cultural knowledge” (50). While the effects of diverse research teams and cultural competence training are challenging to quantify, results seemed optimistic: Wallington and colleagues reported that the culturally competent, community-based strategies led to a 62% increase in the enrolment of black participants in the study compared to previous recruitment tactics (38).

#### 3.3.5 Overcoming practical barriers in study design

Sixteen studies mentioned practical barriers to research participation for potential participants (10, 27, 33, 34, 39, 42, 44, 48, 50, 52–54, 57, 58, 61, 64, 67). These barriers include geographic accessibility, that is, the location where research was being conducted were far or difficult to reach (34, 39, 44, 50, 52, 58), or participants had no access to the right transportation (33, 39, 54, 58); and the time, duration, or timing of trials (31, 54, 58, 61), language barriers (57, 64), among other constraints. These practical barriers can be felt disproportionally by ethnic minority populations because they often have a lower socioeconomic status compared to their White counterparts (68).

For issues related to the geographic accessibility of the research location, different solutions were offered, which focused either on bringing the research site closer to potential participants or facilitating transportation. Wallington and colleagues (38) moved the site from where research and outreach were being conducted to two community-based offices in the medically underserved neighbourhoods where they hoped to recruit for the study. The locations were picked with input from two community advisory boards and were both well-connected to bus and metro lines, as well as being within walking distance for many study participants. To facilitate transportation to the research site, a solution offered by one study was to pay for transportation expenses (27, 30, 56, 60) while colleagues organised transportation to the churches that functioned as project sites in their most successful recruitment arm (33).

## 4 DISCUSSION

This scoping literature review has highlighted several ways to include groups often underrepresented in cancer ED research. It draws on studies published between 2002 and April 2022 in anglophone countries – primarily the USA, that have empirically tested interventions to enhance participant diversity. This review demonstrates that in-person interactions, whether by the research team or designated (peer) recruiters, are important in engaging participants from minoritised groups (33, 44, 67). The importance of face-to-face interactions may be linked to building trust: mechanisms include getting to know the research team in person and having a two-way dialogue to address particular reservations of potential research participants (69, 70). Gren and colleagues (27) found that for the recruitment of minoritised populations, in-person ‘community outreach’ (e.g., recruitment in churches, seminars, and health fairs) was an important addition to direct mail communication, as direct mail alone failed to convince people from minoritised groups to participate in research. Existing literature suggests several locations where such face-to-face interactions could occur, as well as forms of interaction (such as educational workshops and mobile screening services) and aspects to consider when communicating with potential research participants (e.g., culturally tailored- messages).

Another valuable insight from this review is the importance of developing study protocols and recruitment strategies in collaboration with people from the communities researchers hope to recruit from. The engagement strategies discussed in this review were virtually all developed with at least some input from researchers, participants, or other advisors who belong to an underrepresented group. Some studies chose community-based participatory approaches and developed protocols and outreach strategies through collaboration or co-creation with individuals or organisations representing minoritised groups (27, 44, 50). These approaches communicate respect for and attention to minoritised groups researchers want to engage in cancer ED research; they have also been shown to increase statistically positive outcomes in a recent systematic review (71).

This review also documents that combinations of strategies for involving participants from underrepresented groups are necessary. Each recruitment and engagement strategy successfully reaches some groups but excludes others. For example, Sadler and colleagues (45) note that the Black women they recruited through the cosmetologist’s salons are women who can afford such services. Similarly, Leach, Schoenberg, and Hatcher (58) found that, though they conducted research in a rural area characterised by low education levels and high breast cancer mortality, the women they got in touch with through outreach efforts tended to enjoy a higher socioeconomic status and were up to date on breast cancer screening. In other words, even when successful overall, strategies to invite underrepresented groups to participate in cancer ED research tend to exclude specific marginalised subgroups. This suggests that various engagement and recruitment strategies should be combined to reach a wide range of potential participants, and researchers should pay careful attention to their study designs to address the concerns of these (sub)groups. Again, community-based-participatory approaches can help research teams identify these barriers accurately.

This scoping review documents a plethora of barriers and issues in cancer ED research and biomedical research more generally, experienced particularly by members of minoritised groups. These include a lack of trust in researchers, a lack of medical literacy, and insufficient cultural competence in research staff. Although we offer tools to address some of these issues and barriers within a single study or project scope, addressing these issues requires a long-term, collective effort from research institutions and healthcare organisations more generality. Together, these efforts contribute to a more significant shift in biomedical research culture that (re)builds trust between researchers and potential research participants, especially those from minoritised backgrounds. To build trust, relationships between researchers and research participants need to change from appearing extractive to feeling mutually beneficial and collaborative (8). The engagement of minoritised groups in research and inclusive study designs are just one part of this process; increased education and career opportunities in cancer research for people from minoritised backgrounds (72, 73), input from minoritised groups at the policy level, and improved ethnicity coding in healthcare (74) are other essential elements.

This targeted scoping review has limitations that need to be considered. The searches in PubMed and PsycINFO were executed exclusively in English. While many of the manuscripts discussed in this review have tested interventions that could be applied in a variety of contexts and countries, they are based on healthcare systems, socio-economic conditions, and racialised societal relations that may be unique to their own countries, which in turn affect the perceived barriers to cancer ED research.

In addition, the search terms used for this review leave out studies that do not mention recruitment strategies in their title or abstract. Therefore, we may have missed publications for which recruitment strategies are not a focus of the publication, but which are nonetheless described and evaluated in the body of the text.

Finally, this review only includes those studies that have tested or qualitatively evaluated interventions to increase participation or engagement of underserved groups, which means the timescale of these studies is limited: projects that aim to address the historical mistrust of some groups towards cancer research and foster a culture of trust between researchers and research subjects are more challenging to evaluate since they take much longer to bear fruit (75).

Future trials may be necessary to test specific interventions to ascertain their effectiveness in engaging underrepresented groups compared to ‘traditional’ or known recruitment and engagement strategies. Importantly, no intervention will work for all, so evaluations will have to pay careful attention to the particularities of each underrepresented group (76).

In addition, future studies are needed to test the potential for contemporary communications methods to recruit participants from underrepresented groups into cancer ED research. Tested approaches using direct mail and radio commercials may be irrelevant in many societies today, whereas various social media platforms have shown some promise to reach potential cancer ED research participants (77).

Lastly, this review focused on Black and Latinx groups, whereas other (ethnic) minorities received less attention, such as sexual minorities and people with disabilities or co-morbidities. Further developing and standardising recruitment strategies may not lead to the inclusion of *all* societal groups in cancer ED research, as every strategy has its own blind spot. Future research could focus on more neglected groups. These efforts will continue enhancing the diversity of participant research cohorts and contribute to a general shift in research culture and the relationships between researchers and participants.

## 5 CONCLUSION

This scoping literature review has highlighted empirically tested tools that research teams can employ to improve participation rates of groups often underrepresented in cancer ED research. Combinations of these methods can help overcome the perceived barriers to participation in cancer research that mainly affect people without a cancer diagnosis from these minoritised groups. Not only will these methods lead to more representative cancer ED studies that benefit all of society; the highlighted approaches also contribute to a more significant shift in research culture towards less extractive and more trusting relationships between researchers and the public.

## Supplementary Material

Supplementary Files

This is a list of supplementary fi les associated with this preprint. Click to download.

• 08.29.24Scopinglitreviewfi naltable.xlsx

## Figures and Tables

**Figure 7:**
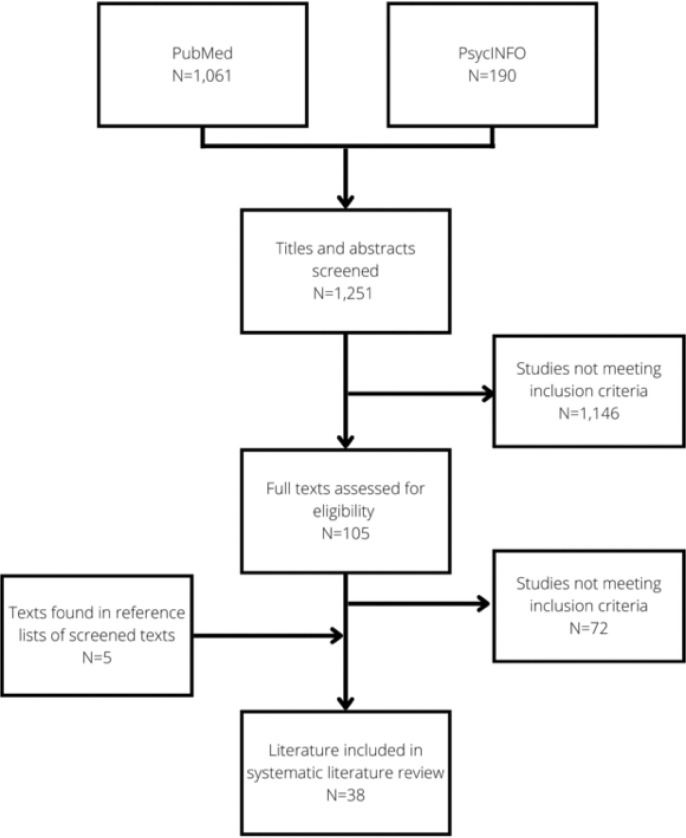
Flow chart of the review inclusion/exclusion process.

**Table 2 -:** Barriers to research participation identified in included studies.

Identified barriers to research participation	Frequency (n)
Mistrust	21
Lack of knowledge	14
Lack of trial awareness	12
Geographic accessibility	8
Lack of cultural competency in research/recruitment	7
Financial burden of participation	4
Time burden of participation	4
Fear of research procedures	4
Concerns about confidentiality/privacy	4
Stigma/misconceptions about participating in research	4
Group not approached enough for research	3
Practical barriers (general)	3
Uninsured/underinsured	2
Fear of uncovering health problems	2
Discrimination	2
Language barriers	2
Limited access to health services	2
Co-morbidities	1

## Data Availability

Data is provided within the manuscript or supplementary information files
